# Clinical presentation and cytokine production abnormalities in a cohort of patients carrying NLRP12 gene variants

**DOI:** 10.1186/1546-0096-12-S1-P71

**Published:** 2014-09-17

**Authors:** Antonella Insalaco, Luigi Raganelli, Manuela Pardeo, Virginia Messia, Denise Pires Marafon, Francesca Romana Lepri, Elisa Pisaneschi, Claudia Bracaglia, Lidia Meli, Valeria Gerloni, Rebecca Nicolai, Elisabetta Cortis, Fabrizio De Benedetti, Giusi Prencipe

**Affiliations:** 1 Division of Rheumatology, Department of Pediatric Medicine, IRCCS Ospedale Pediatrico Bambino Gesù, Italy; 2 Cytogenetics, IRCCS Ospedale Pediatrico Bambino Gesù, Rome, Italy; 3 Pediatric Rheumatology Unit, Department of Rheumatology, Istituto Ortopedico G.Pini University of Milan, Milan, Italy; 4 Division of Pediatric, Santa Maria della Stella Hospital, Orvieto, Italy

## Introduction

The NLRP12 related autoinflammatory disorder (NLRP12-RD) is a rare autosomal dominant disease,caused by mutations in the NLRP12 gene.Clinical manifestations are extremely heterogeneous.At present,only few cases have been described.Patients occasionally required treatment with steroids and NSAIDS for short periods.Treatment with Anakinra induced an initial good response,that appears to decrease over time.

## Objectives

To describe clinical features and inflammatory response of a cohort of patients carrying different NLRP12 variants,some of which not yet described as being associated with NLRP12-RD.

## Methods

Twelve caucasian patients(6 males)carrying NLRP12 variants were identified.Blood samples obtained from 9/12 NLRP12 patients and from 7 active Juvenile idiopathic arthritis(JIA) patients were stimulated ex vivo with 1 mg/ml of Zymosan for 22h.Whole blood RNA analysis was also performed,using a human immune array(TaqMan® Human Immune Array from Applied Biosystems),containing 92 genes typically involved in the immune response.

## Results

The median age at symptoms onset was 11,4 months(IQR 4,6–35,2)and the median of disease duration was 6,8 years(IQR 4,1–11).Sequencing of NLRP12 gene in the 12 patients revealed 5 heterozygous mutations:F402L(n=6),G448A(n=1),H304Y(n=1),R1030G(n=1) and G39V(n=1).Two patients were homozygous for NLRP12 variants:F402L and G39V.In 6/12 variants of NLRP3 were also found:Q703K(n=4) and V198M(n=2).All patients had symptoms consistent with a recurrent inflammatory syndrome:11/12 presented recurrent episodes of skin lesions,11/12 arthralgia,10/12 recurrent fever episodes,8/12 arthritis,10/12 headache,11/12 fatigue,5/12 conjunctivitis,7/12 recurrent abdominal pain and lymphadenopathy,5/12 oral aphthosis,4/12 thoracic pain and 2/12 sensorineural deafness. During the attacks 5/12 patients showed increased acute phase reactants.In 5/12 patients anakinra was administered because of the severity of phenotype and the persistence of elevated acute phase reactants.In 2 of these 5 patients lack of efficacy led withdrawal of anakinra and introduction of tocilizumab with good response.In vitro cytokine release studies,performed in 9 patients,showed that the production of IL-6 and TNF-a was significantly higher in patients carrying the NLRP12 variants compared to patients with JIA (IL-6:2841±1682 ng/ml and 1496±982.4 ng/ml versus 498.8±338.7 ng/ml and 226.6±111.8 ng/ml respectively;p=0.0002 and p=0.007)and even higher in homozygous patients;no significant difference in IL-1β production was found(2134 ± 1026 ng/ml versus 1527±930.3 ng/ml, p=0.29).Whole blood RNA samples collected from 5 NLRP12 patients were compared to 6 whole blood RNA samples collected from healthy controls comparable for age.At basal level,we did not find significant differences in the expression of 92 genes evaluated.

## Conclusion

Our data in vitro and in vivo suggest that these NLRP12 variants are pathogenic.The role played by the concomitant presence of the NLRP3 variants remains to be clarified,though an effect in modifying the disease phenotype cannot be excluded.Our data also confirm the clinical and functional heterogeneity of NLRP12 related disorder,a condition often misunderstood.Furthermore,although the small number of patients treated,our data suggest that inhibition of IL-6 may be effective in NLRP12-related disorder.

## Disclosure of interest

None declared.

